# Successful Treatment With Evocalcet Against Familial Hypocalciuric Hypercalcemia Type 3 (FHH3) Identified by *AP2S1* Gene Mutation (p.Arg15Leu)

**DOI:** 10.1155/crie/9514578

**Published:** 2025-01-23

**Authors:** Ai Chida, Yutaka Hasegawa, Toshie Segawa, Daisuke Yamabe, Hirotaka Yan, Yusuke Chiba, Hiraku Chiba, Hirofumi Kinno, Tomoyasu Oda, Yoshihiko Takahashi, Koji Nata, Yasushi Ishigaki

**Affiliations:** ^1^Division of Diabetes, Metabolism and Endocrinology, Department of Internal Medicine, Iwate Medical University 028-3695, Yahaba, Japan; ^2^Department of Orthopaedic Surgery, Iwate Medical University 028-3695, Yahaba, Japan; ^3^Division of Medical Biochemistry, School of Pharmacy, Iwate Medical University 028-3694, Yahaba, Japan

**Keywords:** adaptor-related protein complex 2 subunit sigma 1 (AP2S1), calcium-sensing receptor (CaSR), evocalcet, familial hypocalciuric hypercalcemia type 3 (FHH3), hypercalcemia

## Abstract

**Background:** Familial hypocalciuric hypercalcemia type 3 (FHH3) is a rare hereditary disorder caused by a heterozygous *AP2S1* gene mutation, characterized by hypocalciuria and hypercalcemia due to impaired intracellular signal transduction of calcium (Ca)-sensing receptors (CaSRs). All affected patients harbored a heterozygous missense mutation at the Arg15 residue of the encoded AP2σ1.

**Case Presentation:** A 21-year-old female was referred to our hospital with hypercalcemia and reduced bone mineral density (BMD) detected during a preoperative examination for scoliosis surgery. She had a developmental disorder and exhibited hypocalciuria on urinalysis. Genetic testing revealed a heterozygous *AP2S1* gene mutation (p.Arg15Leu), and the patient was diagnosed with FHH3. In the present case, we investigated the effects of evocalcet, a newly approved CaSR agonist. Treatment with evocalcet gradually decreased and normalized the serum Ca level, and promoted improvements in bone metabolism, without serious adverse events.

**Conclusion:** Evocalcet may be a promising therapeutic candidate for symptomatic FHH3.

## 1. Introduction

Familial hypocalciuric hypercalcemia (FHH) is a rare autosomal dominant genetic disease caused by abnormalities in calcium (Ca)-sensing receptors (CaSRs) and their signal transduction [1]. They are classified into three different types depending on the location of the responsible genes. Type 1 is caused by loss-of-function mutations of *CASR* encoding CaSR; type 2 is by mutations of *GNA11* encoding G-protein subunit *α*11 (G*α*11), and type 3 is by mutations of *AP2S1* encoding adapter-related protein complex 2 subunit sigma 1 (AP2*σ*1). These phenotypes are characterized by a persistent increase in the serum Ca level, inappropriately low urinary Ca excretion, and a nonsuppressed serum parathyroid hormone (PTH) level [2, 3]. Regarding FHH type 3 (FHH3), *AP2S1* gene mutations have been reported to date; all patients have heterozygous missense substitutions of AP2*σ*2 at the Arg15 residue (Arg15Cys, Arg15His, and Arg15Leu) [4], which causes a decrease in CaSR-mediated intracellular signaling [4]. Among FHH3 cases, stronger phenotypes, such as marked hypercalcemia, reduced bone density, comorbid cognitive, and behavioral disorders, have been reported with Arg15Leu mutations [1, 5–7].

As for the treatment, the majority of FHH cases do not require any treatment. As there are several reports of symptoms and morbidities related to hypercalcemia, effective treatments have been proposed for symptomatic cases [3]. Calcimimetic drugs, such as cinacalcet, have been reported to be effective against the symptoms and morbidity related to FHH [1, 8–10], including FHH3 [11–13]. Recently, a newly approved oral calcimimetic drug, evocalcet, has shown equivalent efficacy to cinacalcet and a lower incidence of gastrointestinal-related adverse events [14–16]. Therefore, evocalcet treatment for FHH3 could be considered an appropriate alternative treatment.

Herein, we describe a rare case of FHH3 patient with a heterozygous *AP2S1* gene mutation (p.Arg15Leu). Treatment with evocalcet normalized the serum Ca level and contributed to the improvement of bone metabolism markers without obvious adverse events. To the best of our knowledge, this is the first report on the efficacy of evocalcet in FHH3.

## 2. Case Presentation

A 21-year-old female was referred to our clinic as hypercalcemia was detected on preoperative examination for scoliosis surgery. In the medical history, she was diagnosed with a pervasive developmental disorder and had hypercalcemia (serum Ca 12.6 mg/dL) at the age of five. However, the serum Ca level had never been measured while a patient at a psychiatric clinic. After being diagnosed with a pervasive developmental disorder during childhood, she attended a vocational school. Currently, she works in the hotel service business. She had been prescribed lithium carbonate for bipolar disorder since the age of 13 years and diagnosed with scoliosis at the age of 15 years. There was no family history of hypercalcemia, developmental disorders, or scoliosis.

**Figure 1:**
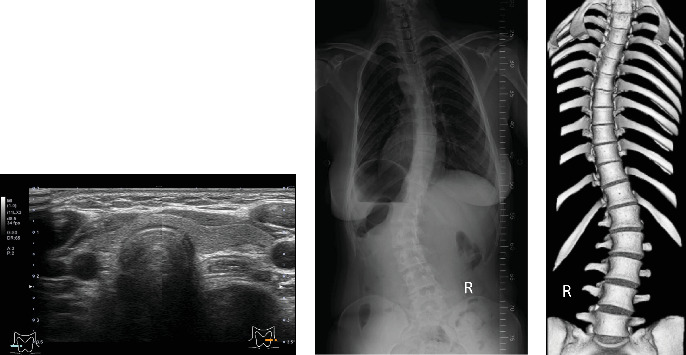
Clinical images of the patient with FHH3. (A) An ultrasonographic image of the neck. (B) The posterior view of the spinal radiograph showing scoliosis. (C) The three-dimensional computed tomography image of the patient. FHH3, Familial hypocalciuric hypercalcemia type 3.

Her height and weight were 167.0 cm and 58.1 kg, respectively, and blood pressure was 116/64 mmHg. Thyroid ultrasonography revealed no enlarged parathyroid glands (Figure 1A). Abdominal computed tomography (CT) imaging revealed no calcified lesions in the urinary tract. Scoliosis was observed on radiography and three-dimensional CT (Figure 1B,C). The Cobb angles on the proximal thoracic, main thoracic, and thoracolumbar/lumbar radiographs were 4.4°, 35.6°, and 41.2°, respectively. Lumbar spine bone mineral density (BMD) was 0.687 g/cm^2^, 67% of the young adult mean (YAM), and *Z*-score, −3.1, which were low values despite the young age. The serum Ca level was high (12.5 mg/dL), although the intact PTH level was not suppressed (50.7 pg/mL) (Table 1). The serum magnesium level was slightly elevated (2.4 mg/dL). Measurement of total 24-h urine Ca revealed lower urinary Ca excretion (60 mg/day) and fractional excretion of Ca (FECa = 0.33%). As the patient had hypercalcemia since childhood and exhibited hypocalciuria with an unsuppressed parathyroid PTH level, FHH was suspected. After obtaining informed consent from the patient for genetic analysis, next-generation sequencing was performed. A heterozygous missense mutation (c.44G >T, p.Arg15Leu) was detected in the *AP2S1* gene, and the patient was diagnosed with FHH3. The asymptomatic parents did not consent to genetic analysis.

Owing to persistent hypercalcemia and concomitant low bone density, treatment of hypercalcemia was considered to be necessary. Previous reports have reported the efficacy of cinacalcet, which acts as an allosteric modulator on CaSRs, against FHH3 [8, 11, 12]. Notably, evocalcet, a newly approved calcimetric drug, has a mechanistic action similar to cinacalcet and is effective in improving bioavailability and reducing gastrointestinal tract-related adverse events [15, 17]. Therefore, this was considered to be an appropriate or superior treatment in this case. After obtaining approval from the hospital's Pharmaceutical Affairs Committee and obtaining patient consent, treatment with evocalcet was initiated.

**Figure 2:**
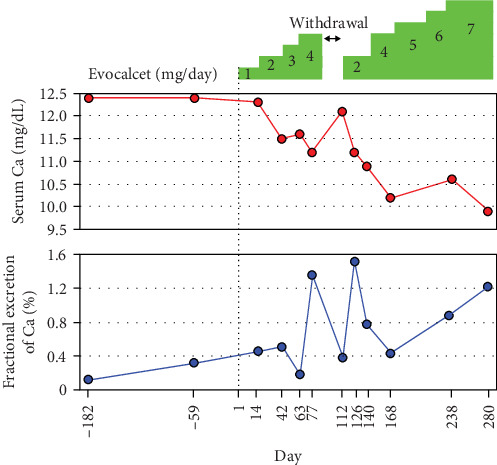
Changes in the serum level of calcium and fractional excretion of Ca before and during treatment with evocalcet. Ca, calcium.

After initiating treatment with evocalcet, the serum Ca level gradually decreased (Figure 2). The patient had indeterminate complaints about fingernails, so the treatment was discontinued for 14 days. The symptoms of the complaints improved during follow-up, and restarting the medication relowered the elevated serum Ca level. With a gradual increase in medication dose, oral administration of evocalcet (7 mg/day) normalized the serum Ca level (Figure 2). In parallel with the change in the serum Ca level, the FECa increased (Figure 2), whereas the level of intact PTH remained within normal limits. Although no apparent improvement in BMD was observed during this short-term treatment, the fasting level of total procollagen type I N-terminal propeptide (P1NP), a bone formation marker, increased from 83.5 to 98.5 ng/mL (Table 2). In contrast, the level of tartrate-resistant acid phosphatase 5b (TRACP-5b), a bone resorption marker, decreased from 337 to 303 mU/dL (Table 2). These data indicated that evocalcet is highly effective against FHH3 by normalizing the Ca level and promoting bone metabolism.

## 3. Discussion

FHH3 is a rare autosomal dominant genetic disease caused by a loss-of-function mutation in the *AP2S1* gene. Due to a decrease in CaSR signal transduction, it typically exhibits hypercalcemia, hypocalciuria, hypermagnesemia, and hypophosphatemia. Despite the high level of serum Ca, the PTH level is normal or mildly elevated due to the decreased sensitivity of CaSR [3, 18]. Compared to FHH type 1, patients with FHH3 present with a higher plasma Ca concentration [5, 19]. Among the three variants of FHH3 (p.Arg15Cys, p.Arg15His, and p.Arg15Leu), this patient has a genetic variant of the *AP2S1* gene at Arg15 residue (p.Arg15Leu). Previously, it has been reported that patients with the FHH3 variant (p.Arg15Leu) have marked hypercalcemia, which causes problems such as pancreatitis and decreased BMD and is associated with a high incidence of neurodevelopmental disorders. Regarding FHH3, it has been reported that ~60% of patients with the p.Arg15Leu mutation have a learning disability [5, 20]. This patient had cognitive impairment and attended vocational school during childhood. This case is a sporadic case of FHH3, and there is no family history of cognitive impairment. Although the detailed mechanism of cognitive impairment due to *AP2S1* gene mutation is still unclear, exposure to marked hypercalcemia from infancy or childhood may lead to neurological developmental disability [21]. In addition, impaired CaSR signaling and transduction may impair neurological development [22]. Indeed, expression of mutant AP2*σ*2 subunits within the brain may directly influence neurological development and mediate receptor trafficking within neuronal synapses of the hippocampus [5, 23]. Furthermore, expression of the AP2 complex regulates synaptic vesicle recycling in neurons in a Ca-independent manner [24].

To date, the efficacy of treatment with calcimimetic drugs, that is, CaSR modulators, such as cinacalcet, has been reported in several symptomatic cases of FHH [11, 13, 25, 26]. A newly approved CaSR modulator, evocalcet, has been shown to be as effective as cinacalcet and has fewer gastrointestinal-related adverse effects [15, 16, 27]. Unlike cinacalcet, evocalcet does not affect the activity of cytochrome P450 enzymes, suggesting that it is a better alternative with higher bioavailability [28, 29]. The efficacy and safety of long-term administration of evocalcet have also been demonstrated [27, 30]. Therefore, we concluded that it was more appropriate to administer evocalcet rather than cinacalcet to this patient. As a result of evocalcet administration, the serum Ca level gradually declined and returned to a normal value. In this case, no obvious side effects, such as upper gastrointestinal disorders, were observed after treatment. Notably, 14 days after drug withdrawal, the elevated serum Ca level returned to a high level, confirming its reversibility. In fact, the correction of hypercalcemia alleviated the patient's anxiety and enabled her to obtain and continue work as a hotel staff member.

The patient also exhibited reduced bone density and scoliosis. As more than 50% of patients with FHH3 exhibit decreased bone density, CaSR signals play important roles in bone formation, and patients are likely to develop osteoporosis or osteomalacia [1, 19]. Before the initiation of evocalcet treatment, this young patient presented with a decreased BMD in the lumbar vertebrae of −3.1SD (67% YAM). One year of evocalcet treatment slightly decreased the BMD in the lumbar vertebrae to −3.5SD (62.0% YAM). In contrast, a bone formation marker (total P1NP) increased, whereas a bone resorption marker (TRACP-5b) decreased after evocalcet treatment. These data clearly demonstrate the efficacy of evocalcet on bone mineral metabolism in this patient. Although there have been no reports of patients with FHH3 with scoliosis, mice lacking the CaSR exhibit bone defects and delayed cartilage and growth plate development [31, 32]. These reports suggest that defects in Ca signal transduction due to the mutant AP2*σ*1 protein may cause scoliosis. We expect that the precise mechanism will be elucidated in future studies.

## 4. Conclusion

We experienced a case in which evocalcet, a CaSR agonist, was effective against FHH3, identified by an *AP2S1* gene mutation (c.44G >T, p.Arg15Leu). Evocalcet is considered to be an effective treatment for FHH3; however, long-term surveillance is required to assess its safety and monitor the serum Ca level and bone mineral metabolism.

## Figures and Tables

**Table 1:** Summary of laboratory data.

	Component	Result	Reference range
Biochemistry	TP (g/dL)	7.4	6.6–8.1
	Alb (g/dL)	4.5	4.1–5.1
	Na (mEq/L)	138	138–145
	K (mEq/L)	4.1	3.6–4.8
	Cl (mEq/L)	107	101–108
	Ca (mg/dL)	12.4	8.8–10.1
	Pi (mg/dL)	2.4	2.7–4.6
	Mg (mg/dL)	2.4	1.8–2.3
	CK (U/L)	48	59–248
	Intact-PTH (pg/mL)	50.7	10.0–65.0
	PTHrP (pmol/L)	<1.0	0–1.1
	25-Hydroxyvitamin D (ng/mL)	21.5	30.0–100.0
	1,25-Dihydroxyvitamin D (pg/mL)	87.6	20.0–60.0
	Total P1NP (ng/mL)	83.5	16.8–70.1
	TRACP-5b (mU/dL)	337	120–420
	TSH (μg/mL)	1.56	0.5–5.0
	FT4 (ng/dL)	1.44	0.9–1.7
	FT3 (pg/mL)	2.81	2.3–4.0
	GH (ng/mL)	0.15	0.13–9.88
	IGF-1 (ng/mL)	252	161–425
	LH (mIU/mL)	12.53	1.13–14.22
	FSH (mIU/mL)	6.3	1.47–8.49
	PRL (ng/mL)	24.6	4.91–29.32

Urine	Protein	—	—
	Glucose	—	—
	RBC	—	—
	Ketone	—	—
	Ca (mg/day)	60	150–290
	Pi (mg/day)	300	500–1000
	Cre (mg/day)	700	500–1500
	FECa (%)	0.33	—

Abbreviations: Alb, albumin; CK, creatine kinase; Cre, creatinine; FECa, fractional excretion of Ca; FSH, follicle-stimulating hormone; FT3, free triiodothyronine; FT4, free thyroxine; GH, growth hormone; IGF-1, insulin-like growth factor; LH, luteinizing hormone; PRL, prolactin; PTH, parathyroid hormone; PTHrP, parathyroid hormone-related protein; RBC, red blood cells; Total P1NP, total type 1 procollagen-N-propeptide; TP, total protein; TRACP-5b, tartrate-resistant acid phosphatase-5b; TSH, thyroid stimulating hormone.

**Table 2:** Data of BMD and bone metabolism markers before and during treatment with evocalcet.

	First visit	After treatment initiation (Day 238)
BMD of L4 (g/cm^2^)	0.687	0.639
Total P1NP (ng/mL)	83.5	98.5
TRACP-5b (mU/dL)	337	303
25-(OH)-vitamin D (ng/mL)	21.5	18
1,25-(OH)_2_-vitamin D (pg/mL)	87.6	183

Abbreviations: BMD, bone mineral density; Total P1NP, total type 1 procollagen-N-propeptide; TRACP-5b, tartrate-resistant acid phosphatase-5b.

## Data Availability

The data that support the findings of this study are available from the corresponding author upon reasonable request.
